# Adipose Tissue-Derived Mesenchymal Stem/Stromal Cells and Their Contribution to Angiogenic Processes in Tissue Regeneration

**DOI:** 10.3390/ijms23052425

**Published:** 2022-02-22

**Authors:** Agnieszka Krawczenko, Aleksandra Klimczak

**Affiliations:** Laboratory of Biology of Stem and Neoplastic Cells, Hirszfeld Institute of Immunology and Experimental Therapy, Polish Academy of Sciences, R. Weigla 12, 53-114 Wroclaw, Poland; agnieszka.krawczenko@hirszfeld.pl

**Keywords:** mesenchymal stem cells, MSCs secretome, angiogenesis, tissue regeneration

## Abstract

Mesenchymal stem/stromal cells (MSCs) are widely described in the context of their regenerative and immunomodulatory activity. MSCs are isolated from various tissues and organs. The most frequently described sources are bone marrow and adipose tissue. As stem cells, MSCs are able to differentiate into other cell lineages, but they are usually reported with respect to their paracrine potential. In this review, we focus on MSCs derived from adipose tissue (AT-MSCs) and their secretome in regeneration processes. Special attention is given to the contribution of AT-MSCs and their derivatives to angiogenic processes described mainly in the context of angiogenic dysfunction. Finally, we present clinical trials registered to date that concern the application of AT-MSCs and their secretome in various medical conditions.

## 1. Introduction

Among the various types of adult stem cells, mesenchymal stem cells (MSCs) seem to be the most frequently described population in the context of tissue regeneration. The term “mesenchymal stem cells” was proposed by Caplan 30 years ago to describe a type of adult stem cells capable of multipotential differentiation into the osteogenic, chondrogenic, and adipogenic lineage [1]. Since then, extensive research on MSCs has focused on their biological behavior in tissue homeostasis and tissue repair. Currently, the term MSCs is used to describe a heterogeneous population of multipotential stem/progenitor cells commonly referred to as mesenchymal stem cells, multipotential stromal cells, mesenchymal stromal cells, and mesenchymal progenitor cells, isolated from different tissue sources [2].

MSCs originating from the third germ layer are multipotent stromal cells present throughout the body that can differentiate into a variety of cell types. A population of MSCs should meet the minimal criteria described by the International Society for Cellular Therapy in 2006 [3], including adhesion to a plastic surface in standard culture conditions, a co-expression of the CD73, CD90, and CD105 antigens, and the ability to differentiate into adipo-, chondro-, and osteogenic lineages, as well as a lack of the expression of the hematopoietic markers CD34, CD45, CD14, and CD79α and the co-stimulatory molecules CD40, CD80, and CD86. Cells meeting these criteria are isolated from different tissues and organs. Although the best-known sources are bone marrow and adipose tissue, MSCs are also present in the skin, heart muscle, skeletal muscles, umbilical cord, peripheral blood, lung, and others tissues [4,5]. Mesenchymal stem/stromal cells are widely described in terms of their immunomodulatory, anti-inflammatory, and regenerative properties. These therapeutic effects apply not only to the cells, but also to their cellular derivatives, i.e., the extracellular vesicles (EVs): exosomes (Exo) and microvesicles (MVs). In this review, we focus on the role of mesenchymal stem/stromal cells of adipose tissue origin (AT-MSCs) and their secretome in the regulation of damaged tissue regeneration with emphasis on the angiogenic processes that are necessary for the proper function of regenerated tissues.

## 2. Mesenchymal Stem/Stromal Cells from Adipose Tissue (AT-MSCs)

### 2.1. Biology of AT-MSCs

Alongside bone marrow, adipose tissue is the most commonly used source of mesenchymal stem cells due to its good accessibility and convenience in obtaining, as it is often a byproduct of many cosmetic and medical procedures. AT-MSCs display a fibroblast-like, spindle-shaped morphology and a high proliferation activity. Cultured adipose tissue-derived stromal/stem cells (ASCs), described also as mesenchymal stromal cells (MSCs), which is the term approved by the International Society for Cell Therapy (ISCT) in 2006 [3,6], possess a high multilineage differentiation potential as well as immunomodulatory activity, a feature that is very important in regenerative medicine. Human AT-MSCs were first isolated by Zuk et al. in 2001 [7] after a liposuction procedure. Since then, interest in AT-MSCs has steadily increased, resulting in a growing number of scientific studies on this subject.

**Figure 1:**
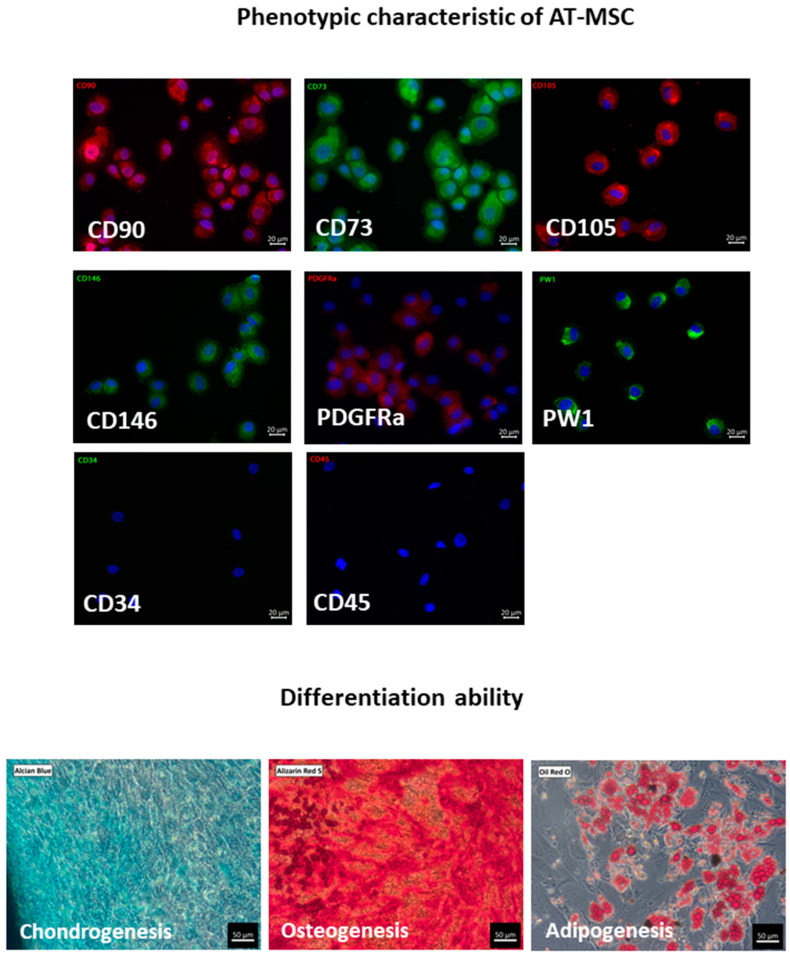
Immunophenotyping and differentiation ability of primary AT-MSCs. Cells are positive for common MSC markers CD90, CD73, CD105 and additionally for CD146, PDGFRa, and PW1. AT-MSCs do not express CD34 and CD45 antigens (immunofluorescence staining, scale bar represents 20 μm). CD90, CD105 and PDGFRa were stained with AlexaFluor 594 (red), CD73, CD146 and PW1 were stained with AlexaFluor 488 (green). Cell nuclei were stained with DAPI. According to the minimal criteria for MSCs, cells are able to differentiate to chondrocytes, osteocytes, and adipocytes as confirmed by staining using Alcian Blue for chondrogenesis, Alizarin Red S for osteogenesis, and Oil Red O for adipogenesis (scale bar represents 50 μm). All pictures come from our own research and are available in the BINWIT open database [11].

Human AT-MSCs have a basic MSC phenotype expressing CD73, CD90, and CD105 as well as HLA ABC markers with a concomitant lack of expression of CD34, CD45, CD14, and HLA DR [3,8]. Additionally, AT-MSCs may also express CD146, PDGFRα, CD44, CD29, and CD13 [9,10]; furthermore, in contrast to bone marrow-derived MSCs (BM-MSCs), they also express the PW1 marker [10]. AT-MSCs are able to differentiate into adipocytes, chondrocytes, and osteoblasts, and therefore fulfill the minimal criteria of MSCs (Figure 1) [3].

The differentiation potential of MSCs is regulated by the expression of the transcription regulators of pluripotency, such as Sox2, Oct4, NANOG, and c-Myc. The expression of these factors in AT-MSCs has been reported by several research groups [10,12,13,14]. In addition to the ability of AT-MSCs to differentiate into classical adipo-, osteo-, and chondrogenic lineages, these cells are also described as being able to differentiate into neural cells, skeletal myocytes, cardiomyocytes, smooth muscle cells, hepatocytes, pancreatic/endocrine cells, and endothelial cells [15,16]; however, the function of the obtained differentiated cells was not always confirmed. In recent years, the regenerative potential associated with MSCs has been described not only in relation to the cells themselves (as possessing the ability to differentiate into other cell types), but also to their derivatives, i.e., the biologically active factors produced by MSCs.

### 2.2. Paracrine Activity and Secretome of AT-MSC

**Figure 2:**
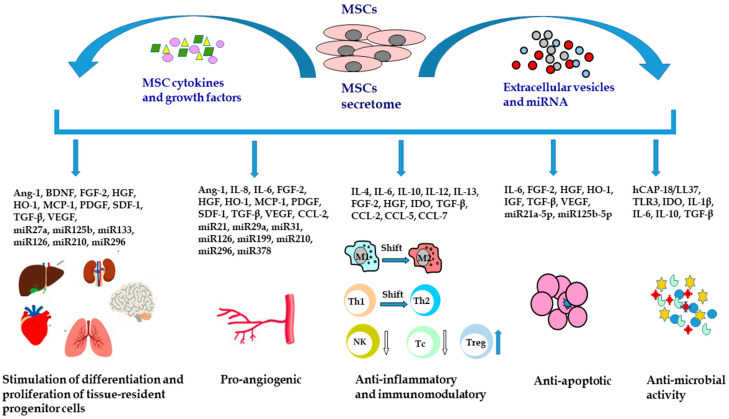
Mesenchymal stem/stromal cells secretome and its therapeutic activity. MSCs can stimulate the differentiation and proliferation of tissue-resident progenitor cells, induce angiogenesis, modulate the inflammatory response, prevent cell apoptosis, and exert antimicrobial activity. Ang-1, angiopoietin 1; BDNF, brain-derived neurotrophic factor; CCL-2, C-C motif chemokine ligand 2; CCL-5, C-C motif chemokine ligand 5; CCL-7, C-C motif chemokine ligand 7; FGF-2, fibroblast growth factor 2; hCAP18/LL37, human cationic antimicrobial protein; HGF, hepatocyte growth factor; HO-1, heme oxygenase-1; IDO, indoleamine 2,3-dioxygenase; IGF, insulin-like growth factor; IL, interleukin; M1, M1 macrophages; M2, M2 macrophages; MCP-1, monocyte chemoattractant protein-1; miR, microRNA; MSCs, mesenchymal stem cells; NK, natural killer cells; PDGF, platelet derived growth factor; SDF-1, stromal cell-derived factor 1; Tc, cytotoxic T cells; Th1, T helper cells type 1; Th2, T helper cells type 2; TGF-β, transforming growth factor β; TLR3, toll-like receptor 3; Treg, regulatory T cells; VEGF, vascular endothelial growth factor.

In regenerative medicine, the therapeutic effects of MSCs are not limited to direct cell-to-cell interactions. MSCs secrete a broad spectrum of biologically active factors, including growth factors, cytokines, chemokines, cell adhesion molecules, lipid mediators, hormones, exosomes, microvesicles, and other regulatory molecules such as miRNA. The secretome is an essential component involved in the therapeutic effect of MSCs, primarily derived from its ability to stimulate cell proliferation, new blood vessels formation, immunomodulatory properties, and anti-apoptotic and anti-microbial activity (Figure 2).

Despite the differences in the secretome profile of MSCs isolated from different tissue sources, functional analysis revealed that the MSC secretome has similar characteristics promoting cell migration and inhibiting cell apoptosis [17]. Furthermore, many studies have confirmed that MSCs secrete a number of antimicrobial peptides/proteins (AMPs) and thus show direct antimicrobial activity [18,19,20,21,22]. In recent years, the secretome of AT-MSC has been studied thoroughly, especially in the context of cell-free therapy for wound treatment [23,24,25]. Special attention (including in the studies conducted by the authors of this paper) is paid to a group of secreted factors with an angiogenic potential, i.e., VEGF A, VEGF D, angiogenin, and IL-8, because AT-MSCs are one of the richest cellular producers of these cytokines among other analyzed MSCs derived from different sources [10,26,27,28].

The secretome of AT-MSCs varies depending on microenvironmental conditions and in vitro cell culture mode. The most common stimuli that modulate the composition of MSCs are hypoxia and inflammation [29]. Tissue damage is very often accompanied by hypoxia, which makes hypoxia the most potent factor affecting AT-MSCs and their secretome at the tissue injury site. Hypoxia augmented the production of cytokines and bioactive factors responsible for angiogenic properties (VEGF, FGF, SDF-1, IGF, and others), growth factors (TGF-β1, TGF-β2), and chemokines (RANTES) [28,30]. Other stimulatory factors affecting the composition of the AT-MSC secretome are inflammatory stimuli, such as interferon γ (IFN-γ), interleukin 6 (IL-6), or lipopolysaccharide (LPS). In response to these molecules, AT-MSCs produce a number of anti-inflammatory factors, including the IDO enzyme, HGF, and TGF-β [31,32,33]. Several studies describe the anti-apoptotic and antibacterial activity of AT-MSCs [18,22,34,35,36,37], which was also confirmed in our own investigations [38].

## 3. AT-MSC-Derived Extracellular Vesicles (EVs): Exosomes (Exo) and Microvesicles (MVs)

An important part of the secretory activity of MSCs are EVs, a heterogeneous population of cell-derived membrane nonreplicating particles that includes Exo, MVs, and apoptotic bodies [39]. The EVs are released by all normal and apoptotic cells. In the case of neoplastic cells, tumor cells release EVs named oncosomes [40,41]. However, in the context of their potential application in regenerative medicine, only the first two groups released by MSCs, i.e., Exo and MVs, are tested due to their biological content.

### 3.1. Mode of Formation

Based on the size and mechanism of release, EVs are divided into three main groups: Exo, MVs, and apoptotic bodies. The exosomes are the smallest EVs in size, ranging from 30 to 150 nm. They form inside multivesicular bodies and are then released from the cell through exocytosis. The second group contains MVs ranging from 100 to 1000 nm that are released directly from the cell membrane. The largest ones (1000–5000 nm) are apoptotic bodies, which form during the apoptotic cell death [39].

### 3.2. Biological Content

EVs transport a large number of biologically active molecules, including proteins, lipids, DNA, RNA, and regulatory molecules such as microRNA. Because of the different ways in which EVs, Exo, MVs, and apoptotic bodies form, they differ in their cargo. For example, Exo carry specific proteins (tetraspanins and Alix) that are used as markers to distinguish them from other EVs, whereas MVs contain proteins that are specific to the cells they originate from [39]. The biological content of EVs obtained in in vitro and ex vivo cultures depends on different factors, including cell type, culture method, and isolation procedure. The same cell type may secrete different types of EVs having different cargo depending on the mode of formation (e.g., Exo vs. MVs), environmental conditions (e.g., oxygen concentration), or other stimuli (e.g., apoptosis) [42]. The protein content of EVs reflects the metabolic activity of the parental cell, and the activation pathway determines the number and cargo of the formed EVs [43]. Furthermore, different EV subgroups can be isolated with varying efficiency using multiple techniques, which include sequential centrifugation, differential ultracentrifugation, density gradient centrifugation (sucrose or iodixanol gradients), filtration, and size-exclusion chromatography [42,44].

### 3.3. Intercellular Communication

Because EVs are produced by all types of the cells, they are involved in intercellular communication throughout the body, acting both in the autocrine and paracrine manner, and are systemic in the whole body, being released into body fluids. As a result, EVs participate in a number of physiological and pathological processes, including tumor metastasis. A growing number of reports also show that circulating EVs can serve as biomarkers in various diseases, such as cardiovascular diseases, cardiometabolic diseases, nonalcoholic fatty liver disease, and various types of cancer [45,46,47,48,49]. There are different modes of transfer of the EV cargo into the recipient cells, including direct contact with membrane fusion, receptor-ligand interaction, or endocytosis [50]. The components of EVs are then released into the cytoplasm of the target cell and induce various biological processes. Consequently, EVs are considered for inclusion in cell-free therapy to provide the benefits of the MSC immunomodulatory activity but without the risk of immune rejection in allogeneic conditions [51].

### 3.4. AT-MSC-Derived Extracellular Vesicles (EVs)

AT-MSCs produce both types of EVs, Exo and MVs. Although some articles clearly distinguish these two groups of vesicles, more often researchers use the term EVs, which covers the heterogeneous population of both studied groups. EVs produced by AT-MSCs contribute to intercellular communication and participate in many physiological and pathological processes, similarly to the widely-described MSCs of bone marrow origin. AT-MSC-EVs reduce the inflammatory response and accelerate tissue regeneration, as well as act as immunosuppressive factors [52,53]. AT-MSC-derived EVs have been described as biomarkers and/or therapeutic factors in neurological disorders [54,55], cardiovascular diseases [56], liver injury [57,58], renal diseases [56], and skin injuries [53,59,60]. However, the best-known and the most often described function of AT-MSC-EVs is related to their regenerative potential in the regulation of anti-inflammatory responses within the inflammatory microenvironment.

## 4. Effect of AT-MSCs on Angiogenic Processes

Angiogenesis is a multistep process of blood vessel formation from existing vessels. This is affected by different factors, most importantly, the vascular endothelial growth factor (VEGF), hypoxia, and nitric oxide concentration.

AT-MSCs produce and secrete many angiogenic factors, as compared to other MSC sources, including VEGF, transforming growth factor β1 (TGF-β1), vascular endothelial growth factor D (VEGF D), angiogenin (ANG), interleukin 8 (IL-8), insulin-like growth factor-1 (IGF-1), growth-regulated protein (GRO), epithelial neutrophil activating peptide (ENA-78), and platelet derived growth factor-BB (PDGF-BB). The presence of angiogenic factors has been confirmed at the mRNA and protein level, both in a soluble form in a conditioned medium and in EVs [26,27,28,38]. Moreover, a number of regulatory pro-angiogenic miRNAs have been detected. This abundant production of angiogenic factors strongly indicates that AT-MSCs and their derivatives participate in angiogenic processes during tissue regeneration. However, the beneficial effect of AT-MSCs on angiogenesis may be related not only with their paracrine activity but also with their differentiation potential towards pericyte-like cells, which play an important role in stabilization of the vessel wall and prevent vascular leakage. These observations performed in vitro both in normal culture conditions and in high glucose concentration medium, to mimic the changed microenvironment of a diabetic eye, revealed that pericyte-like differentiation of human AT-MSCs can serve as a possible therapeutic option in patients of diabetic retinopathy to replace irreversibly lost pericytes [61,62]. A very recent studies of Beloglazova et al. (2022) shows evidence that AT-MSCs support endothelial cell network through the uPA pathway (uPA-uPAR/VEGFR2/integrin/NOTCH). These findings can help to understand and potentially control aberrant angiogenesis [63]. Bi et al. demonstrated that the human stromal vascular fraction (SVF) obtained from adipose tissue as well as AT-MSCs accelerate wound healing in diabetic mice and improve the formation of the capillary structure of human umbilical vein endothelial cells (HUVEC) [64]. Interestingly, a conditioned medium obtained from senescent AT-MSCs attenuates the angiogenic potential of these cells [65]. Trinh et al. described a study on human AT-MSC-MVs in wound healing in vitro and in vivo [66]. The authors showed that AT-MSCs obtained from diabetic patients and transfected with MVs isolated from the AT-MSCs of healthy individuals changed their gene expression profile. The transfection improved diabetic AT-MSC functions by altering the expression of miR29c and miR150 and up-regulating the expression of genes associated with cell migration, survival, inflammation, and angiogenesis (SDF-1, CXCR4, CXCR7, CCL2, and ANGPTL4). The transfection of diabetic AT-MSCs with AT-MSC-MVs obtained from healthy individuals improved the migration ability of the transfected diabetic AT-MSCs in vitro and wound healing ability in a flap mouse model. A study conducted by Zomer et al. evaluated the application of MSCs derived from the dermis and adipose tissue in cutaneous wound healing in vitro [67]. The authors showed that both AT-MSCs and dermis MSCs could be applied for skin wound healing, with the dermis MSCs showing an increased healing ability and their conditioned media exerting a greater paracrine effect during the wound healing process than the AT-MSCs.

A recent review by Alonso-Alonso et al. [68] described different molecules reported to date in AT-MSC-EVs. They showed that a total of 591 proteins and 604 miRNAs have been detected in human AT-MSC-EVs. Among the proteins involved in angiogenic processes, the platelet-derived growth factor (PDGF) was studied in vitro and in vivo by Lopatina et al. The authors showed that PDGF stimulated the secretion of EVs, changed their protein composition, and enhanced their angiogenic potential [69]. Similar studies with TNFα stimulation of AT-MSCs showed an increased mRNA expression of pro-angiogenic factors (FGF-2, VEGF, IL-8, and MCP-1), inflammatory cytokines (IL-1b and IL-6), proteases (MMPs and uPA), and the adhesion molecule ICAM-1. The protein production of VEGF, IL-8, MCP-1, and ICAM-1 and the ability of AT-MSCs to promote microvessel growth in a fibrin gel assay was also enhanced [70]. Zhong et al. investigated the angiogenic factors secreted by AT-MSCs and found that GDNF is a key molecule stimulating the formation of the capillary network in a HUVEC model [71]. Interestingly, this effect was independent from VEGF activity. A study conducted by Pu et al. described the use of AT-MSCs and the conditioned medium (CM) obtained from these cells to enhance neovascularization and skin survival after an ischemia/reperfusion (I/R) injury. The authors demonstrated that the AT-MSC-CM and AT-MSC-Exo increased tube formation, possibly through an increased expression and secretion of IL-6 [72].

Other researchers investigated the effect of hypoxic conditions on the angiogenic properties of AT-MSC-CM. Hypoxia promoted the angiogenic properties of AT-MSC-CM through an increased expression of HIF1α and its downstream protein vascular endothelial growth factor A (VEGF A), as shown in a tube formation assay [73]. Hypoxic conditions also enhanced the production of angiogenin (ANG) and interleukin 8 (IL-8), which are known for their proangiogenic activity [74]. Moreover, EVs secreted by AT-MSCs cultured in hypoxic conditions led to an increase in the production of VEGF in HUVEC endothelial cells [75] and significantly increased vascular tube formation [76].

An important role in angiogenesis is also played by nucleic acids, including mRNA and small non-coding RNAs, especially miRNAs. In AT-MSC-EVs 84 mRNAs and 489 different miRNAs were recognized. Many of the detected mRNAs coded proteins involved in angiogenesis, including VEGF A, IL-8, IL-6, IGF1, ANG, FGF2, PDGFA, KDR, and CDH5 (VE-cadherin) [68]. A large number of miRNAs detected in AT-MSC-EVs included both pro- (e.g., let7b, let7g, miR126, miR199, miR21, miR29a, and miR31) and antiangiogenic (e.g., miR125, miR221, miR222, miR34a, and miR92a) microRNA. The expression of miRNA regulating angiogenesis in AT-MSCs and AT-MSC-MVs was described by Huang et al. [77]. The authors demonstrated that AT-MSC-MVs were rich in miRNAs related to angiogenesis, including two members of the let-7 family. AT-MSC-MVs can be taken up by HUVEC endothelial cells to promote the migratory and invasive ability of endothelial cells. Our own experiment also showed that MVs derived from immortalized human AT-MSCs (HATMSC1) that contained pro-angiogenic miRNAs, i.e., miR126, miR296, miR378 and miR210, enhanced angiogenesis, as observed in vitro with a tube formation assay [28].

## 5. AT-MSCs and Their Derivatives: Contribution to Regenerative Processes

Different research groups analyzed the potential of MSCs in the regeneration of different tissues. However, results similar to the effect of cell application were obtained following the application of conditioned media harvested from AT-MSCs containing many biologically active factors and EVs released from these cells. Indeed, AT-MSCs and their secretome were demonstrated to contribute to skin injuries (wound healing), muscle damage, nerve regeneration, bone regeneration, and lung tissue regeneration. Examples of this activity are presented below.

### 5.1. Preclinical Studies on AT-MSCs in Tissue Regeneration

The most frequently described regenerative activity connected with the application of AT-MSCs and their secretome concerns wound healing. AT-MSCs secrete a plethora of regenerative growth factors, cytokines, chemokines, and immune mediators that affect processes related to wound healing, e.g., inflammation, angiogenesis, and extracellular matrix remodeling.

A recent study conducted by Pomatto et al. [78] showed that factors produced by AT-MSCs and encapsulated in EVs preferentially promote wound healing, in contrast to BM-MSC-EVs. An analysis of AT-MSC-EV cargo revealed the presence of molecules mainly associated with angiogenesis, including both proteins and miRNA. Moreover, AT-MSC-EVs had a beneficial effect on the cells that participate in wound healing, i.e., fibroblasts, keratinocytes, and endothelial cells, improving their migration and facilitating angiogenic processes, which was shown in in vitro experiments on human fibroblasts, keratinocytes, and endothelial cells, and further confirmed in a murine model of diabetic wounds [78]. These results match our own experience, as we showed that AT-MSC-MVs carry proteins and miRNAs that support and facilitate proangiogenic processes during wound healing [28]. Moreover, the obtained results also proved that the CM from cultured AT-MSCs significantly enhanced the proliferation of fibroblasts, endothelial cells, and keratinocytes in vitro in a chronic wound model [27,38]. Accelerated wound healing was also described by Zhao et al. [79], who used Exo from human adipose-derived mesenchymal stem cells to treat diabetic cutaneous wounds in a mouse model. They observed accelerated wound closure, re-epithelialization, enhanced collagen production, angiogenesis, cell proliferation, inhibited apoptosis, and reduced inflammation.

AT-MSCs and the factors released by these cells also contributed to the regeneration of damaged muscles. De La Garza-Rodea et al. showed that AT-MSCs contribute to skeletal muscle regeneration in cardiotoxin-injured mice. Moreover, they demonstrated that the transplantation of human AT-MSCs into the cardiotoxin-damaged tibialis anterior muscles of immunodeficient mice caused the creation of a larger amount of hybrid myofibers than treatment with BM-MSCs or synovial membrane-derived MSCs [80]. The regenerative effects of AT-MSCs, as with BM-MSCs, were also described in a rat skeletal muscle laceration injury. Two studies conducted by Abd Elaziz (2019) and Moussa (2020) showed the effectiveness of rat AT-MSC delivery into an injured muscle. In both cases, increased myotube formation was observed after the AT-MSC treatment [81,82]. Another example involved the use of the CM obtained from AT-MSCs in an in vitro study on Duchenne muscular dystrophy (DMD). Assoni et al. demonstrated that the AT-MSC-derived CM modulated the apoptosis of dystrophic myoblasts and enhanced cell migration and proliferation [83]. The regenerative effect of human AT-MSC-EVs and the soluble fraction of the MSC secretome on muscle injury was confirmed by Mitchell et al. [84].

AT-MSCs have also been studied with respect to bone regeneration processes. The osteogenic and angiogenic potential of human AT-MSCs and BM-MSCs was investigated in vitro and in vivo. The results showed that AT-MSCs were unable to form ectopic bone when applied to nude mice, but exhibited an enhanced angiogenic potential in vitro compared to BM-MSCs [85]. Another study described the enhanced ability of Exo derived from human AT-MSCs to regenerate bone after a pretreatment of the cells with the tumor necrosis factor-alpha (TNF-α), as was evidenced in vitro through an increased the proliferation and osteogenic differentiation of human primary osteoblastic cells [86].

There are several reports describing the contribution of AT-MSCs and their secretome in neural regeneration. Even though AT-MSCs are capable of neurogenic differentiation, most studies did not confirm their direct differentiation into neurons. Instead, the role of paracrine-secreted factors in the repair of damaged nerves is emphasized. Most of the studies were carried out with rodents, in particular, a rat model, with AT-MSCs or their EVs derived from murine, canine, or human adipose tissue [87,88,89]. A study conducted by Bucan et al. [90] strongly suggested that EVs from rat AT-MSCs promoted nerve regeneration and neurite growth after a sciatic nerve crush injury. Administration of human AT-MSCs to the sciatic nerve injury accelerated functional recovery in mice. This effect is hypothesized to originate from the ability of AT-MSCs to induce the production of the glial-derived neurotrophic factor (GDNF) by murine Schwann cells in vivo. Additionally, AT-MSCs were able to produce and secrete factors such as the brain-derived neurotrophic factor (BDNF), bFGF, and IGF-1 [91]. Brini et al. analyzed the therapeutic effect of the CM obtained from human AT-MSCs (AT-MSC-CM) in comparison with the AT-MSC treatment of neuropathic pain in a mouse model of type 1 diabetes [92]. The results demonstrated that an intravenous injection of either AT-MSCs or AT-MSC-CM reverted neuropathic hypersensitivity, reduced inflammation, and prevented the loss of skin innervation. An excellent review concerning the role of AT-MSCs and their secretome in peripheral nerve regeneration processes was presented very recently by Sumarwoto et al. [93].

### 5.2. Clinical Application of AT-MSCs and Their Derivatives in Tissue Regeneration

AT-MSCs and their secretome are described not only in in vitro studies, but also in many clinical trials. A recent publication by Carstens et al. [94] demonstrated the safety and beneficial effect of an adipose stromal vascular fraction injection, containing both endothelial progenitor cells and MSCs, into the wound bed and periphery of patients with large diabetic foot ulcers (>3 cm in diameter). Over a one-year observation, significant wound healing was observed, leading to complete healing of the wound in the majority of cases.

A total of 269 clinical trials are registered at ClinicalTrials.gov [95] (accessed on 28 January 2022) that use the key term adipose mesenchymal stem cells, 164 of which are active, recruiting, terminated, or completed. The medical conditions in which AT-MSCs or their derivatives are applied include ischemic disorders (ischemia reperfusion injury, ischemic stroke, and critical limb ischemia), cardiac and vascular diseases (heart failure, pulmonary hypertension, and Buerger’s disease), tendon, ligament, and joint injuries (ligament injury, knee osteoarthritis, knee osteoarthrosis, tendon injury, and degenerative arthritis), skin damage (diabetic foot ulcer, burn, trophic ulcer, and therapy of scars), brain injuries, nerve system and neurological disorders (multiple system atrophy-Parkinson variant, severe brain injury, secondary progressive multiple sclerosis, Alzheimer disease, amyotrophic lateral sclerosis, spinal cord injuries, cerebral palsy, cerebellar ataxia, brachial plexus neuropathies, Parkinson’s disease, traumatic brain injury, and refractory epilepsy), autoimmune diseases (systemic sclerosis, psoriasis, systemic lupus erythematosus, chronic autoimmune urticaria, and rheumatoid arthritis), type 1 and 2 diabetes mellitus, pulmonary infections, immunological disorders (discordant immunological response in HIV-infected subjects; acute, chronic, and expanded graft versus host disease), and other conditions. Table 1 presents selected examples of clinical trials using AT-MSCs and their secretome in diseases related to angiogenic dysfunction.

Since the beginning of the SARS-CoV-2 pandemic, MSCs have also been considered as supportive therapy in severe pneumonia caused by the coronavirus disease (COVID-19) [106,107]. The virus binds the angiotensin-converting enzyme 2 (ACE2) receptor present on target cells and utilizes membrane bound cell transmembrane protease serine 2 (TMPRSS2) to enter the cells. A recent study of Generali et al. (2022) showed that although ACE2 receptor and TMPRSS2 are expressed on the surface of many cells, MSCs derived from three main human tissue sources (adipose tissue, umbilical cord Wharton’s jelly, and bone marrow) do not express ACE2 but TMPRSS2 [108]. These findings are important considering future MSC-based therapies in COVID-19 disease. Interestingly and notably, except for Wharton jelly-derived MSCs, which are the most often used in clinical applications in patients with COVID-19, there are two completed clinical trials using AT-MSCs. One of them (NCT04349631) evaluated the safety and efficacy of an infusion of autologous AT-MSCs in supporting immunity against COVID-19, conducted in subjects with no signs of COVID-19. No results have been published yet. Another study (phase II) assessing the efficacy of AT-MSCs in supporting immunity against the coronavirus disease (NCT04348435) has been completed very recently. Nine other clinical trials using allogeneic AT-MSCs and two trials using autologous AT-MSCs for the prevention and treatment of the coronavirus disease or treatment of patients with long-haul symptoms after COVID-19 are ongoing. There are also two completed clinical trials exploring the safety and efficiency of AT-MSC exosomes used as aerosol inhalation (NCT04313647) and for the treatment of patients hospitalized with coronavirus pneumonia (NCT04276987). The first study (NCT04313647) proved that aerosol inhalation of AT-MSCs-derived exosomes is safe and does not cause serious adverse events in healthy volunteers [109]. The second one (NCT04276987) was a pilot clinical trial performed to explore the safety and efficiency of aerosol inhalation of the exosomes derived from allogenic adipose MSCs (MSCs-Exo) in patients with severe novel coronavirus pneumonia (NCP). The results are not yet publicly available.

There are two more trials in which AT-MSC exosomes were applied in other diseases. The first one (NCT04388982) is an ongoing phase I/Ⅱ clinical trial that explores the safety and efficacy of allogenic adipose MSCs-Exos in the treatment of mild to moderate dementia caused by Alzheimer’s disease. The second one (NCT04544215) concerns the application of AT-MSC exosomes as aerosol inhalation in pulmonary infections caused by Gram-negative bacilli resistant to carbapenems. Another trial concerns the use of AT-MSC secretome (either the complete conditioned medium or EVs) for the treatment of osteoarthritis and/or for articular regeneration (NCT04223622). The estimated completion date of the study is December 2022. Two more trials using the secretome of AT-MSCs are registered; one of which uses an AT-MSC conditioned medium in the treatment of ligament injury (NCT04889963), whereas the second study uses the AT-MSC secretome together with umbilical cord MSCs in the treatment of patients with multiple system atrophy, Parkinson variant (NCT04876326).

## 6. Conclusions and Future Perspectives

AT-MSCs and their secretome represent a promising therapeutic tool in regenerative medicine due to their unique properties especially in supporting angiogenesis. Results obtained in experimental and clinical studies suggest that AT-MSCs and AT-MSC derivatives, EVs, and CM could be considered as factors supporting muscular, neural, and cutaneous repair and regeneration. The beneficial effects of AT-MSCs and their secretome are related to their ability to deliver a wide range of bioactive molecules, including proteins, nucleic acids, exosomes, microvesicles, and regulatory elements such as microRNA to the target cells, which results in enhanced tissue repair and regeneration.

## Figures and Tables

**Table 1:** Application of AT-MSCs and/or their secretome in diseases related to angiogenic disorders.

Study Number/Status	Type of Disease	Type of Therapy	Patients	Results	Ref.
Not reported Completed	Nonhealing diabetic foot ulcers (>3 cm in diameter)	Local injections of autologous adipose-derived stromal vascular fraction (SVF) cells (EPCs and MSCs), phase 1 study; injection into the target foot of a total dose of 30 × 10^6^ SVF cells	63 patients with type 2 diabetes and underlying microangiopathy	Improved ulcer healing: closure response rates among evaluable patients between 86% and 93% at the 6- and 12-month endpoints; changes in the vascular bed beneath the ulcer and structural characteristics of the arteries supplying the foot	[94]
KB/27/2015 Bioethics committee at the Regional Specialist Hospital, Research and Development Center in Wroclaw, Poland Completed	Chronic venous stasis ulcers	Subcutaneous administration to the tissues surrounding the ulcers and under the ulcer bed of autologous AT-MSCs (3.0 × 10^5^ to 2.3 × 10^7^ cells)	11 patients (12 ulcers)	Improvement in clinical condition observed in 75% of ulcers; complete healing occurred in 25% of ulcers	[96]
NCT04746599 Recruiting ClinicalTrials.gov	Critical limb ischemia	Autologous fat grafting	20 participants	No results posted	
NCT04661644 Recruiting ClinicalTrials.gov	Critical limb ischemia	Clusters of adipose-derived mesenchymal stem cells (dose: 1 × 10^7^ cells/1 mL/vial, phase 1; and 1 × 10^8^ cells/1 mL/vial, phase 2), phase 1/2a clinical trial	20 participants	No results posted	
NCT04466007 Recruiting ClinicalTrials.gov	Critical ischemia of the lower limbs in diabetic patients without the possibility of revascularization	Allogeneic mesenchymal stromal cells derived from adipose tissue administered intramuscularly (low and high doses)	90 participants	No results posted	
NCT03968198 Recruiting ClinicalTrials.gov	Critical limb ischemia and peripheral artery disease	Autologous intramuscular administration of adipose tissue-derived mesenchymal stromal/stem cells (ASCs), phase 2 study	43 participants	No results posted	
NCT01824069 Completed ClinicalTrials.gov	Nonrevascularizable critical ischemia of the lower limbs	Intramuscular injection of autologous adult mesenchymal stem cells derived from adipose tissue (1 × 10^6^/kg), phase 1 and 2a study	10 participants	7 patients were followed-up after the treatment for 1 year (phase 1b study). A statistically significant improvement in health-related quality of life in the post-treatment period was observed. An ankle-brachial index and clinical behavior of the limb improved during the follow-up.	[97]
NCT01745744 Completed ClinicalTrials.gov	Critical chronic ischemic syndrome of the lower limb in nondiabetic patients	Infusion of mesenchymal stem cells derived from adipose tissue administered intraarterially: 0.5 × 10^6^ cells/kg of patient weight and 1 × 10^6^ cells/kg of patient weight, phase 2 study	33 participants	No results posted	
NCT01663376 Completed	Critical Limb Ischemia	Intramuscular injection of autologous adipose derived mesenchymal stem cells. Dose: 1 × 10^8^–3 × 10^8^ cells	20 participants	Autologous AT-MSC implantation effectively increases blood flow. Above 66% of patients with non-healing ulcers experienced ulcer healing, only in the cases with an initially necrotic foot, no observable tissue regeneration occurred. There was clinical improvement in 100% of patients with a diabetic foot (3 patients) and in 58.3% of patients with Buerger’s Disease (7 patients)	[98]
NCT01302015 Completed ClinicalTrials.gov	Buerger’s disease (thromboangiitis obliterans)	RNL-Vascostem (autologous adipose tissue-derived mesenchymal stem cells) dosage: intramuscular infusion, 5 × 10^6^ cells/kg	15 participants		
NCT04569409 Recruiting ClinicalTrials.gov	Diabetic foot ulcer	Application of a hydrogel sheet (ALLO-ASC-DFU) containing allogenic adipose-derived mesenchymal stem cells to diabetic grade 2 foot ulcer, phase 3 study	104 patients	No results available yet	
NCT04497805 Recruiting ClinicalTrials.gov	Diabetic foot ulcer	Application of a hydrogel sheet (ALLO-ASC-DFU) containing allogenic mesenchymal stem cells to diabetic grade 2 foot ulcer, phase 2 study	64 participants	No results available yet	
NCT04457037 Completed ClinicalTrials.gov	Trophic ulcer	Patients with trophic ulcers received standard treatment and autologous adipose-derived mesenchymal stem cells	18 participants	No results available yet	
NCT03276312 Completed ClinicalTrials.gov	Minor amputations of diabetic foot	Lipogems–local injection of autologous micro-fragmented adipose tissue	112 participants	After 6 months, 80% of the micro-fragmented adipose tissue-treated feet healed and 20% failed compared to the control group. A significant improvement in terms of physical health-related quality of life and a significant reduction of the hospital length of stay was reported.	[99,100]
NCT03183648 Active, not recruiting ClinicalTrials.gov	Burn	Application of a hydrogel sheet (ALLO-ASC-DFU) containing allogenic adipose-derived mesenchymal stem cells	30 participants	No results available yet	
NCT04280003 Recruiting ClinicalTrials.gov	Ischemic stroke	Intravenous treatment with allogenic adipose tissue-derived stem cells in a single dose of one million cells per kg, phase 2 study	30 participants	No results available yet	[101]
NCT02387723 Completed ClinicalTrials.gov	Heart failure	Patients with heart failure treated with culture-expanded adipose tissue-derived mesenchymal stem cells from healthy donors. The cells were injected directly into the myocardium	10 participants	Four out of ten patients developed donor-specific de novo HLA class I antibodies, and two other patients had donor-specific antibodies at baseline. None of the patients had any clinical symptoms or changes in biochemical or inflammatory parameters. The cardiac function tended to improve after AD MSC treatment at 6-month follow-up.	[102]
NCT01678534 Completed ClinicalTrials.gov	Ischemic Stroke	Intravenous treatment with allogeneic stem cells from adipose tissue, phase 2 study	19 participants	No results posted	[103]
NCT01449032 Completed ClinicalTrials.gov	Chronic ischemic heart disease (coronary artery disease, CAD)	Intramyocardial injections of autologous VEGF-A165-stimulated adipose-derived stem cells (ASCs), phase 2 study	60 participants	Intramyocardially delivered ASC treatment was safe but did not improve exercise capacity compared to placebo in a pilot study. After a 3-year follow-up, patients receiving ASCs had improved cardiac symptoms and unchanged exercise capacity, in contrast to deterioration in the placebo group	[104,105]
NCT04388761 Recruiting ClinicalTrials.gov	Ischemia reperfusion injury in patients with a kidney allograft	AMSC treatment via direct injection into the kidney parenchyma and intra-arterial infusion, phase 2 study	15 participants	No results posted	
NCT01257776 Completed ClinicalTrials.gov	Critical limb ischemia (CLI) in diabetic patients	Intra-arterial administration of autologous adipose-derived mesenchymal stem cells, phase 1 study	33 participants	No results posted	
NCT03865394 Completed ClinicalTrials.gov	Chronic wounds in diabetic foot syndrome	Application of allogeneic adipose-derived mesenchymal stem cells in fibrin gel	46 participants	No results posted	
NCT03183726 Completed ClinicalTrials.gov	Diabetic foot ulcer	Application of ALLO-ASC-DFU (a hydrogel sheet containing allogenic adipose-derived mesenchymal stem cells), phase 1 study	4 participants	No results posted	
NCT03754465 Recruiting ClinicalTrials.gov	Diabetic foot ulcer	Application of an ALLO-ASC-DFU sheet to diabetic foot ulcer (a hydrogel sheet containing allogenic adipose-derived mesenchymal stem cells), phase 2 study	44 participants	No results posted	
NCT03370874 Active, not recruiting ClinicalTrials.gov	Diabetic foot ulcer	Application of an ALLO-ASC-DFU sheet to diabetic foot ulcer (a hydrogel sheet containing allogenic adipose-derived mesenchymal stem cells), phase 3 study	164 participants	No results posted	
NCT02394873 Completed ClinicalTrials.gov	Deep second-degree burn wound	Application of an ALLO-ASC-DFU sheet (a hydrogel sheet containing allogenic adipose-derived mesenchymal stem cells), phase 1 study	5 participants	No results posted	
NCT05165459 Recruiting ClinicalTrials.gov	Venous keg ulcer	Venous leg ulcer treatment with adipose SVF (autologous adipose stromal vascular fraction) administered locally into the target ulcer.	10 participants	No results posted	
NCT04569409 Recruiting ClinicalTrials.gov	Diabetic Wagner grade 2 foot ulcers	Application of an ALLO-ASC-DFU sheet (a hydrogel sheet containing allogenic adipose-derived mesenchymal stem cells), phase 3 study	104 participants	No results posted	

## Data Availability

Not applicable.

## Abbreviations

AMPs	antimicrobial peptides/proteins
ANG	angiogenin
ANGPTL4	angiopoietin-related protein 4
Ang-1	angiopoietin 1
ASCs	adipose tissue-derived stromal/stem cells
AT-MSCs	adipose tissue-derived mesenchymal stem cells
BDNF	brain-derived neurotrophic factor
BM-MSCs	bone marrow derived mesenchymal stem cells
CCL2	C-C Motif chemokine ligand 2, MCP-1
CCL5	C-C motif chemokine ligand 5, RANTES
CCL7	C-C Motif chemokine ligand 7
CDH5	VE-cadherin (vascular endothelial cadherin)
CM	conditioned medium
c-Myc	transcription factor c-Myc
CXCR4	C-X-C chemokine receptor type 4
CXCR7	C-X-C chemokine receptor type 7
DMD	Duchenne muscular dystrophy
ENA-78	epithelial neutrophil activating peptide
EPCs	endothelial progenitor cells
EVs	extracellular vesicles
Exo	exosomes
FGF	fibroblast growth factor
GDNF	glial-derived neurotrophic factor
GRO	growth-regulated protein
HATMSC1	human adipose tissue MSCs cell line
hCAP18/LL37	human cationic antimicrobial protein
HGF	hepatocyte growth factor
HIF1α	hypoxia-inducible factor 1-alpha
HLA ABC	human leukocyte antigen, major histocompatibility complex, class I
HLA DR	human leukocyte antigen, major histocompatibility complex, class II
HO-1	heme oxygenase-1
HUVEC	human umbilical vein endothelial cells
ICAM-1	intercellular adhesion molecule 1
IDO	indoleamine 2,3-dioxygenase
IFN-γ	interferon gamma
IGF	insulin-like growth factor
IL	interleukin
KDR	kinase insert domain receptor, FLK1, vascular endothelial growth factor receptor 2
LPS	lipopolysaccharides
MCP-1	monocyte chemoattractant protein-1, CCL2
miR	microRNA
MSCs	mesenchymal stem cells
MMPs	matrix metalloproteinases
MVs	microvesicles
NANOG	homeobox protein NANOG, transcriptional factor
NOTCH	a transmembrane receptor in a signaling pathway
Oct4	octamer-binding transcription factor 4
PDGFA	platelet derived growth factor A
PDGF-BB	platelet derived growth factor-BB
PDGFRα	platelet-derived growth factor receptor A
PW1	Peg3, paternally expressed gene 3, a marker of adult stem cells
RANTES	regulated on activation, normal T cell expressed and secreted protein, CCL5
SDF-1	stromal cell-derived factor 1, C-X-C motif chemokine 12, CXCL12
Sox2	(sex determining region Y)-box 2, transcription factor
SVF	stromal vascular fraction
TGF-β	transforming growth factor β
TLR3	toll-like receptor 3
TNFα	tumor necrosis factor alpha
uPA	urokinase-type plasminogen activator
uPAR	urokinase-type plasminogen activator receptor
VEGF	vascular endothelial growth factor
VEGFR	vascular endothelial growth factor receptor
